# Vibration Analysis of Post-Buckled Thin Film on Compliant Substrates

**DOI:** 10.3390/s20185425

**Published:** 2020-09-22

**Authors:** Xuanqing Fan, Yi Wang, Yuhang Li, Haoran Fu

**Affiliations:** 1 Institute of Solid Mechanics, Beihang University (BUAA), Beijing 100191, China; fanxuanqing@buaa.edu.cn; 2 Aircraft and Propulsion Laboratory, Ningbo Institute of Technology, Beihang University, Ningbo 315800, China; warindy@buaa.edu.cn; 3 Frontier Research Center, Institute of Flexible Electronics Technology of THU. Zhejiang, Jiaxing 314006, China; fuhaoran@ifet-tsinghua.org

**Keywords:** post-buckling, free vibration, thin film, elastic substrate, flexible electronics

## Abstract

Buckling stability of thin films on compliant substrates is universal and essential in stretchable electronics. The dynamic behaviors of this special system are unavoidable when the stretchable electronics are in real applications. In this paper, an analytical model is established to investigate the vibration of post-buckled thin films on a compliant substrate by accounting for the substrate as an elastic foundation. The analytical predictions of natural frequencies and vibration modes of the system are systematically investigated. The results may serve as guidance for the dynamic design of the thin film on compliant substrates to avoid resonance in the noise environment.

## 1. Introduction

As is well known, buckling instability appears in the film/substrate system when subjected to external loading, which is utilized by stretchable electronics to achieve stretchability. Recent advances in the buckling of thin films on a compliant substrate enable high stretchability for stretchable electronics [1,2,3,4,5,6,7,8], which can be used as human vital signs monitors [9,10,11,12,13], sensitive electronic skins [14,15,16], eye-like digital cameras [17,18,19], tunable phase optics [20,21], and tunable optical window [22,23].

In these systems, controlled buckling is generated in thin films deposited onto prestrained substrate after releasing the prestrain in the substrate [24,25,26,27,28,29,30,31,32]. The post-buckling morphology can be global buckling and local buckling with different modulus of substrate [24,25]. Bowden et al. [26] showed the buckling morphology surface within 100 nm–100 μm range in the film. Furthermore, Khang et al. [28] illustrated wave length and amplitude with specific external pre-strains through the experiments and the analytical model. A series of analytical models were developed to precisely predict the post-buckling shape with different material, geometric, and loading conditions [29,30].

However, in operation conditions, the system undergoes complex noise environment with electrical, mechanical, and thermal loadings. The dynamic behavior of the post-buckled system is one of the most important problems to solve for the applications of stretchable electronics. Many investigations on vibration analysis of post-buckling structures have been carried out in recent years [33,34,35,36,37,38,39,40,41,42]. Nayfeh et al. [33] presented an exact solution for the post-buckling configurations of beams, investigates the lowest natural frequencies of vibration around each of the first three buckled configurations beams with various boundary conditions, and discusses the stability of the beam. Emam et al. [34] derived approximate analytical solutions for the nonlinear free vibrations of laminated composite beams in prebuckling and postbuckling. Cristobal et al. [35] investigated dynamic and impact behaviors of glass fiber reinforced polymer composites. Neukirch et al. [36] investigated the vibration of post-buckled rods clamped at both ends. Emam and Nayfeh studied the non-linear response of the buckled beam considering internal resonances [37]. Furthermore, Ansari et al. [38] derived an analytical model to describe the dynamic behaviors of a postbuckled microscale functionally graded (FG) beam with modified coupled stress theory. The response of nonlinear vibration of postbuckled beams has also been investigated numerically [39]. Focusing on stretchable electronics, Wang et al. [41] established an analytical model to predict the dynamic behaviors of buckled thin films considering surface effects, and Wang et al. [42] derived an explicit analytical solution for the vibration mode and the linear natural frequency of a buckled ribbon, which was verified by finite element method (FEM) and experiments. Tseng et al. [43] derived a solution to the linear natural frequency of the first-order vibration mode for a buckled ribbon with fixed ends. The former papers have paid much attention to buckling and dynamic behaviors of thin film. However, they are not directly applicable in solving the vibration problem for flexible electronics, in which the compliant substrates beneath the devices vibrate together with thin films. Currently, there are very scant investigations of analytical solution on the vibration analysis of post-buckled thin films on a compliant substrate. With only the assistance of numerical simulations, it is difficult to understand the mechanical nature of the film/substrate system and is time-consuming in practical device design and optimization.

Therefore, this paper aims to establish an analytical model to illustrate the dynamic behaviors of this special system. However, dynamic loading, temperature change, vibration environment, and other factors affect the mechanical properties of the substrate, which can make the substrate viscoelastic instead of just being simplified as springs. This paper mainly focuses on hyperelastic substrate such as polydimethylsiloxane (PDMS) with small temperature change and low vibration frequency. In such cases, viscoelasticity of the substrate has negligible influence on the vibration analysis. The outline of this paper is as follows. Section 2 illustrates the analytical modeling for buckling analysis, and the vibration analysis is shown in Section 3. Results and discussion are given in Section 4. The main conclusions are presented in Section 5.

## 2. Buckling Analysis

**Figure 1:**
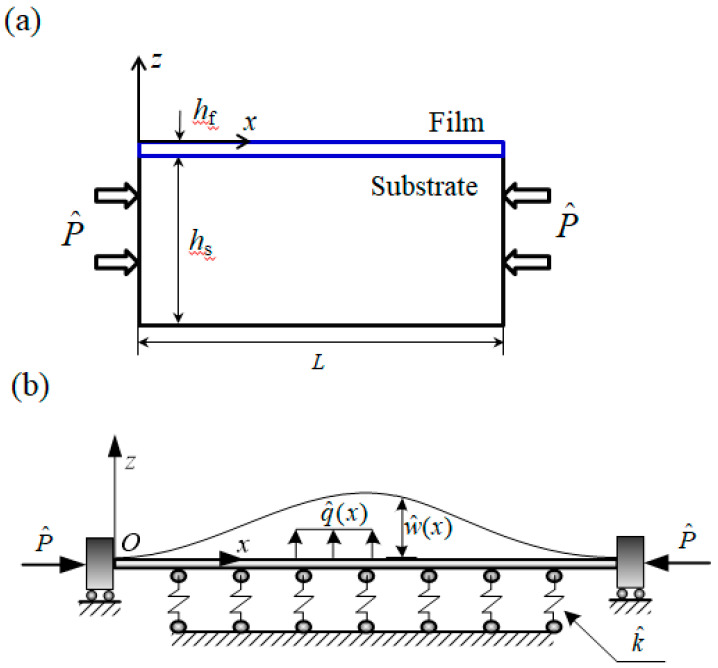
(**a**) Schematic of thin film on a compliant substrate and (**b**) the deformation of the analytical model in the first-order bucking mode with an elastic foundation.

Figure 1a illustrates the thin film with thickness of *h_f_* on top of a compliant substrate with thickness of *h_s_* subjected to a compressive load $\hat{P}$ and non-conservative force $\hat{q}$. Buckling occurs after loading because the thickness of the film is extremely small, which is very similar to a slender beam buckling. In the analytical model, the thin film is considered as Euler–Bernoulli beam with fixed condition at both ends, since the thickness of film *h*_f_ is far less than the length *L* [42]. Planes of the cross sections remain planes after deformation, and the plane of the cross section is still perpendicular to the axis after deformation. It is unnecessary to employ the von Karman plate theory to model the film because it leads to lengthy solutions not convenient for practical use due to the in-plane displacement and the shear traction [44]. A common approximation is to ignore the in-plane displacement and the shear traction [45,46]. The film deformed out of a plane only can be modeled into a beam, which greatly simplifies theoretical analysis [24]. The compliant substrate is considered to be a Winkler elastic foundation [47,48,49,50], whose reaction at any point is proportional to the deflection, with stiffness $\hat{k}$ and deflection of the thin film $\hat{w}$ shown in Figure 1b, where $\hat{w}$ is the function of Cartesian coordinate $\hat{x}$, which is along the axial direction of the thin film.

The kinetic energy of the thin film intra-domain is

(1) $$T=\frac{1}{2}m\int_{0}^{L}{\left(\frac{\partial \hat{w}}{\partial \hat{t}}\right)}^{2}d\hat{x},$$

where *L* is the undeformed length of the film, and *m* is the mass density. The total static potential energy in the film can be expressed as

(2) $$V=\frac{1}{2}EI\int_{0}^{L}{\left(\frac{\partial^{2}\hat{w}}{\partial {\hat{x}}^{2}}\right)}^{2}d\hat{x}-\frac{1}{2}\hat{P}\int_{0}^{L}{\left(\frac{\partial \hat{w}}{\partial \hat{x}}\right)}^{2}d\hat{x}+\frac{EA}{8L}{\left[\int_{0}^{L}{\left(\frac{\partial \hat{w}}{\partial \hat{x}}\right)}^{2}d\hat{x}\right]}^{2}+\frac{1}{2}\int_{0}^{L}\hat{k}{\hat{w}}^{2}d\hat{x},$$

where *E* is Young’s modulus, and *A* and *I* are the area and the moment of inertia of the cross section, respectively. Here, the potential energy due to bending can be expressed as $\frac{1}{2}EI\int_{0}^{L}{\left(\frac{\partial^{2}\hat{w}}{\partial {\hat{x}}^{2}}\right)}^{2}d\hat{x}$. The potential energy due to the axial force P is given by $-\frac{1}{2}\hat{P}\int_{0}^{L}{\left(\frac{\partial \hat{w}}{\partial \hat{x}}\right)}^{2}d\hat{x}$. The potential energy due to the midplane stretching is given by $\frac{EA}{8L}{\left[\int_{0}^{L}{\left(\frac{\partial \hat{w}}{\partial \hat{x}}\right)}^{2}d\hat{x}\right]}^{2}$. The potential energy due to elastic foundation is given by $\frac{1}{2}\int_{0}^{L}\hat{k}{\hat{w}}^{2}d\hat{x}$. Then, the work done by non-conservative force in the film is

(3) $$W_{nc}=\int_{0}^{L}\hat{q}\hat{w}d\hat{x}$$

According to Hamilton’s principle $\int_{{\hat{t}}_{0}}^{{\hat{t}}_{f}}(\delta T-\delta V+\delta W_{nc})d\hat{t}=0$, it can be derived,

(4) $$\begin{array}{l} \int_{{\hat{t}}_{0}}^{{\hat{t}}_{f}}\int_{0}^{L}[-EI\frac{\partial^{4}\hat{w}}{\partial {\hat{x}}^{4}}-\hat{P}\frac{\partial^{2}\hat{w}}{\partial {\hat{x}}^{2}}+\frac{EA}{2L}\frac{\partial^{2}\hat{w}}{\partial {\hat{x}}^{2}}\int_{0}^{L}{\left(\frac{\partial \hat{w}}{\partial \hat{x}}\right)}^{2}d\hat{x}+\hat{q}-\hat{k}\hat{w}-m\frac{\partial^{2}\hat{w}}{\partial {\hat{t}}^{2}}]\delta \hat{w}d\hat{x}d\hat{t}\\ -\int_{{\hat{t}}_{0}}^{{\hat{t}}_{f}}{\left[EI\frac{\partial^{2}\hat{w}}{\partial {\hat{x}}^{2}}\delta (\frac{\partial \hat{w}}{\partial \hat{x}})\right]}_{0}^{L}d\hat{t}+\int_{{\hat{t}}_{0}}^{{\hat{t}}_{f}}{\left[(EI\frac{\partial^{3}\hat{w}}{\partial {\hat{x}}^{3}}+\hat{P}\frac{\partial \hat{w}}{\partial \hat{x}}-\frac{EA}{2L}\frac{\partial \hat{w}}{\partial \hat{x}}\int_{0}^{L}{\left(\frac{\partial \hat{w}}{\partial \hat{x}}\right)}^{2}d\hat{x})\delta \hat{w}\right]}_{0}^{L}d\hat{t}=0 \end{array}$$

where $\hat{t}$ is time. In consideration of the arbitrariness of variation and boundary conditions for fixed support at both ends, the governing equations are obtained:

(5) $$m\frac{\partial^{2}\hat{w}}{\partial {\hat{t}}^{2}}+EI\frac{\partial^{4}\hat{w}}{\partial {\hat{x}}^{4}}+P\frac{\partial^{2}\hat{w}}{\partial {\hat{x}}^{2}}+\hat{k}\hat{w}-\frac{EA}{2L}\frac{\partial^{2}\hat{w}}{\partial {\hat{x}}^{2}}\int_{0}^{L}{\left(\frac{\partial \hat{w}}{\partial \hat{x}}\right)}^{2}d\hat{x}-\hat{q}=0$$

The boundary condition is $\partial \hat{w}/\partial \hat{x}=0$ and $\hat{w}=0$ at $\hat{x}=0$ and $\hat{x}=L$, respectively. For the convenience of deduction, we use the non-dimensional variables as below

(6) $$\begin{array}{l} x=\frac{\hat{x}}{L},\;w=\frac{\hat{w}}{r},\;t=\hat{t}\sqrt{\frac{EI}{mL^{4}}},\\ q=\hat{q}\frac{L^{4}}{rEI},\;P=\frac{\hat{P}L^{2}}{EI},\;k=\hat{k}\frac{L^{4}}{EI} \end{array}$$

where $r=\sqrt{I/A}$ is the radius of gyration of the cross section. Substituting Equation (6) into Equation (5) gives,

(7) $$\ddot{w}+w^{\mathrm{IV}}+Pw^{\prime \prime }+kw-\frac{1}{2}w^{\prime \prime }\int_{0}^{1}{w^{\prime }}^{2}dx-q=0,$$

for fixed condition at both ends,

(8) $$w^{\prime }=0\;\mathrm{and}\;w=0,\;\mathrm{at}(x=0,x=1),$$

where superscript “˙” and “′” stand for the derivation to *t* and *x*, respectively.

In order to solving buckling problem first, the buckling equilibrium equation can be obtained through omitting the time term and transverse load term from Equation (7) and denoting the buckled configuration by Φ(x),

(9) $$\{\begin{array}{c} \Phi^{\mathrm{IV}}+P\Phi^{\prime \prime }-\frac{1}{2}\Phi^{\prime \prime }\int_{0}^{1}{\Phi^{\prime }}^{2}dx+kw=0\\ \Phi^{\prime }=0,\;(x=0,x=1)\\ \Phi^{\prime }=0,\;(x=0,x=1) \end{array}$$

Here, assuming $\sigma^{2}=P-\kappa =P-\frac{1}{2}\int_{0}^{1}{\Phi^{\prime }}^{2}dx$ is a constant, then Equation (9) becomes

(10) $$\left\{ \begin{array}{c} \Phi^{\mathrm{IV}}+\sigma^{2}\Phi^{\prime \prime }+k\Phi =0\\ \Phi^{\prime }=0,\;(x=0,x=1)\\ \Phi =0,\;(x=0,x=1) \end{array}\right.$$

When $\sigma^{4}>4k$, the general solution of Equation (10) can be assumed as

(11) $$\Phi (x)=c_{1}\sin(k_{1}x)+c_{2}\cos(k_{1}x)+c_{3}\sin(k_{2}x)+c_{4}\cos(k_{2}x)$$

where $k_{1}=\sqrt{\left(\sigma^{2}+\sqrt{\sigma^{4}-4k}\right)/2}$ and $k_{2}=\sqrt{\left(\sigma^{2}-\sqrt{\sigma^{4}-4k}\right)/2}$; *c_i_* (*i* = 1,2,3,4) is the coefficient to be determined by the boundary conditions. Substituting Equation (11) into the four boundary conditions of Equation (10) derives

(12) Ac=0,

where,

(13) $$A=\left[\begin{array}{cccc} 0 & 1 & 0 & 1\\ \sin(k_{1}) & \cos(k_{1}) & \sin(k_{2}) & \sin(k_{2})\\ k_{1}\cos(k_{1}) & -k_{1}\sin(k_{1}) & k_{2}\cos(k_{2}) & -k_{2}\sin(k_{2})\\ k_{1} & 0 & k_{2} & 0 \end{array}\right]\;,\;c=\left[\begin{array}{c} c_{1}\\ c_{2}\\ c_{3}\\ c_{4} \end{array}\right]$$

Demanding that the determinant of the coefficient matrix equals zero, σ can be obtained via solving the following characteristic equation,

(14) $$\begin{array}{c} k_{1}k_{2}\cos{\left(k_{1}\right)}^{2}-2k_{1}k_{2}\cos\left(k_{1}\right)\cos\left(k_{2}\right)+k_{1}k_{2}\sin{\left(k_{1}\right)}^{2}-{k_{1}}^{2}\sin\left(k_{1}\right)\sin\left(k_{2}\right)\\ -{k_{2}}^{2}\sin\left(k_{1}\right)\sin\left(k_{2}\right)+k_{1}k_{2}\cos{\left(k_{2}\right)}^{2}+k_{1}k_{2}\sin{\left(k_{2}\right)}^{2}=0 \end{array}$$

It can be seen from the equation $\sigma^{2}=P-\kappa =P-\frac{1}{2}\int_{0}^{1}{\Phi^{\prime }}^{2}dx$ that $P\geq \sigma^{2}$, which means that the critical buckling force is $\sigma^{2}$. We can obtain the value of *c_i_* by solving the Equation (12),

(15) $$\begin{array}{c} c_{1}=-c\frac{k_{2}}{k_{1}}\\ c_{2}=c\frac{k_{1}\sin\left(k_{2}\right)-k_{2}\sin\left(k_{1}\right)}{-k_{1}\cos\left(k_{1}\right)+k_{1}\cos\left(k_{2}\right)},\\ c_{3}=c\\ c_{4}=-c\frac{k_{1}\sin\left(k_{2}\right)-k_{2}\sin\left(k_{1}\right)}{-k_{1}\cos\left(k_{1}\right)+k_{1}\cos\left(k_{2}\right)} \end{array}$$

where *c* is constant. Substituting Equation (15) and Equation (11) into $\sigma^{2}=P-\kappa =P-\frac{1}{2}\int_{0}^{1}{\Phi^{\prime }}^{2}dx$ gives the value of *c*, and the bucking mode Φ(x) can be obtained.

## 3. Vibration Analysis of Post-Buckled System

In order to investigate the vibration problem of the beam near the buckling configuration, we need to introduce a small dynamic displacement ε(x,t),

(16) w(x,t)=Φ(x)+ε(x,t)

Substituting Equation (16) into Equation (7) gives,

(17) $$\varepsilon^{\mathrm{IV}}+\ddot{\varepsilon }+k\dot{\varepsilon }+\sigma^{2}\varepsilon^{\prime \prime }=\frac{1}{2}\Phi^{\prime \prime }\int_{0}^{1}\left(2\Phi^{\prime }\varepsilon^{\prime }+{\varepsilon^{\prime }}^{2}\right)dx+\frac{1}{2}\varepsilon^{\prime \prime }\int_{0}^{1}\left(2\Phi^{\prime }\varepsilon^{\prime }+{\varepsilon^{\prime }}^{2}\right)dx+q,$$

with boundary conditions: ε=0 and $\varepsilon^{\prime }=0$ at x=0,x=1. For the post-buckling linear free vibration problem, the post-buckling vibration equilibrium equation can be derived by omitting the nonlinear term and the transverse load term from Equation (17),

(18) $$\ddot{\varepsilon }+\varepsilon^{\mathrm{IV}}+k\dot{\varepsilon }+\sigma^{2}\varepsilon^{\prime \prime }=\Phi^{\prime \prime }\int_{0}^{1}\Phi^{\prime }\varepsilon^{\prime }dx$$

We assume that ε(x,t) has the following form,

(19) $$\varepsilon (x,t)=\xi (x)e^{i\omega t},$$

where ξ(x) denotes the post-buckling vibrational mode, and *ω* denotes the vibration frequency. Substituting Equation (19) into Equation (18),

(20) $$\begin{array}{c} \xi^{\mathrm{IV}}+\sigma^{2}\xi^{\prime \prime }-\omega^{2}\xi +k\xi =\Phi^{\prime \prime }\int_{0}^{1}\Phi^{Ȃ}\xi^{\prime }dx\\ \xi =0\;\;\left(x=0,1\right)\\ \xi^{\prime }=0\;\;\left(x=0,1\right) \end{array}$$

The general solution of Equation (20) can be expressed as,

(21) $$\xi (x)=\xi_{h}(x)+\xi_{p}(x),$$

where $\xi_{h}(x)$ is the general solution of the equation $\xi^{\mathrm{IV}}+\sigma^{2}\xi^{\prime \prime }-(\omega^{2}-k)\xi =0$, which can be expressed as when $\omega^{2}>k$,

(22) $$\xi_{h}(x)=d_{1}\sin(s_{1}x)+d_{2}\cos(s_{1}x)+d_{3}\sinh(s_{2}x)+d_{4}\cosh(s_{2}x),$$

where *d_i_* are constants, and *s_i_* is

(23) $$s_{1,2}={\left(\pm \frac{1}{2}\sigma^{2}+\frac{1}{2}\sqrt{\sigma^{4}+4(\omega^{2}-k)}\right)}^{\frac{1}{2}}$$

The particular solution $\xi_{p}(x)$ can satisfy Equation (20),

(24) $${\xi_{p}}^{\mathrm{IV}}+\sigma^{2}{\xi_{p}}^{\prime \prime }-\omega^{2}\xi_{p}+k\xi_{p}=\Phi^{\prime \prime }\int_{0}^{1}\Phi^{\prime }{\xi_{h}}^{\prime }dx+\Phi^{\prime \prime }\int_{0}^{1}\Phi^{\prime }{\xi_{p}}^{\prime }dx$$

Assuming that

(25) $$\xi_{p}(x)=d_{5}\Phi^{\prime \prime }$$

Substituting Equation (25) into Equation (24) considering $\Phi^{\mathrm{IV}}+\sigma^{2}\Phi^{\prime \prime }+k\Phi =0$ provides,

(26) $$\int_{0}^{1}\Phi^{\prime }{\xi_{h}}^{\prime }dx+d_{5}\left(\omega^{2}+\int_{0}^{1}\Phi^{\prime }\Phi^{\prime \prime \prime }dx\right)=0$$

As a result, the solution of Equation (20) can be expressed as,

(27) $$\xi (x)=d_{1}\sin(s_{1}x)+d_{2}\cos(s_{1}x)+d_{3}\sinh(s_{2}x)+d_{4}\cosh(s_{2}x)+d_{5}\Phi^{\prime \prime }$$

Then, substituting Φ(x) and ξ(x) into Equation (26) and boundary conditions of Equation (20) gives homogeneous linear equations, which can be rewritten as,

(28) Bd=0,

where

(29) $$B=\left[\begin{array}{ccccc} 0 & 1 & 0 & 1 & -c_{2}k_{1}{}^{2}-c_{4}k_{2}{}^{2}\\ s_{1} & 0 & s_{2} & 0 & -c_{1}k_{1}{}^{3}-c_{3}k_{2}{}^{3}\\ \sin(s_{1}) & \cos(s_{1}) & \sinh(s_{2}) & \cosh(s_{2}) & \alpha_{6}\\ s_{1}\cos(s_{1}) & -s_{1}\sin(s_{1}) & s_{2}\cosh(s_{2}) & s_{2}\sinh(s_{2}) & \alpha_{7}\\ \alpha_{1} & \alpha_{2} & \alpha_{3} & \alpha_{4} & \omega^{2}+\alpha_{5} \end{array}\right]\;,\;d=\left[\begin{array}{c} d_{1}\\ d_{2}\\ d_{3}\\ d_{4}\\ d_{5} \end{array}\right]$$

where *α_i_* are constants as below,

(30) $$\begin{array}{c} \alpha_{1}=\int_{0}^{1}\Phi^{\prime }\sin^{\prime }\left(s_{1}x\right)dx\\ \alpha_{2}=\int_{0}^{1}\Phi^{\prime }\cos^{\prime }\left(s_{1}x\right)dx\\ \alpha_{3}=\int_{0}^{1}\Phi^{\prime }\sinh^{\prime }\left(s_{2}x\right)dx\\ \alpha_{4}=\int_{0}^{1}\Phi^{\prime }\cosh^{\prime }\left(s_{2}x\right)dx\\ \alpha_{5}=\int_{0}^{1}\Phi^{\prime }\Phi^{\prime \prime \prime }dx\\ \alpha_{6}=-c_{1}{k_{1}}^{3}\cos\left(k_{1}\right)+c_{1}{k_{1}}^{3}\sin\left(k_{1}\right)-c_{1}{k_{2}}^{3}\cos\left(k_{2}\right)+c_{1}{k_{2}}^{3}\sin\left(k_{2}\right)\\ \alpha_{7}=-c_{1}{k_{1}}^{3}\cos\left(k_{1}\right)+c_{1}{k_{1}}^{3}\sin\left(k_{1}\right)-c_{1}{k_{2}}^{3}\cos\left(k_{2}\right)+c_{1}{k_{2}}^{3}\sin\left(k_{2}\right) \end{array}$$

Since *d_i_* cannot simultaneously be zero, det(B)=0, which provides the condition to obtain the value of *ω*. Furthermore, the vibrational mode ξ(x) corresponding to natural frequency *ω* can be obtained.

## 4. Results and Discussion

**Figure 2:**
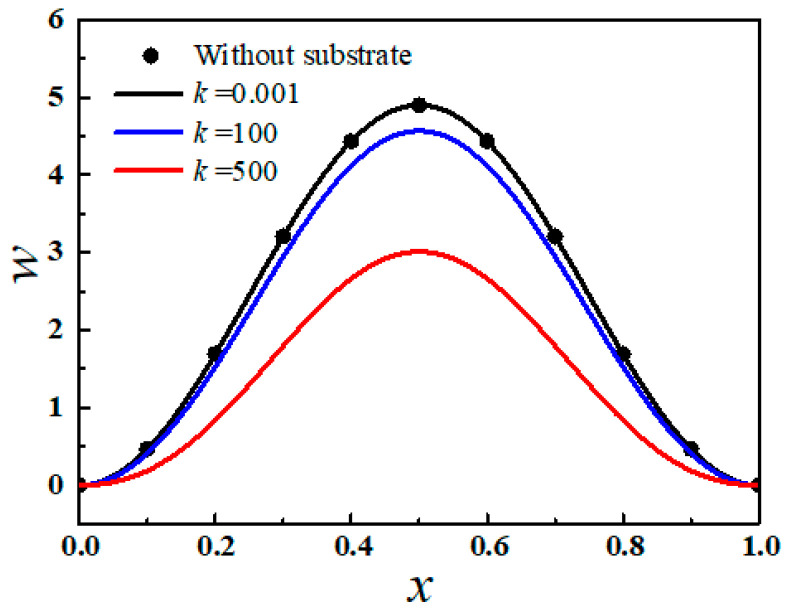
The deflection of first-order buckling mode in the film with different substrate stiffness or without substrate when *P* = 10 × π^2^.

The effective stiffness of substrate *k* can have significant effects on the buckling behaviors of the thin film. Figure 2 shows the deflection of the first-order buckling mode in the films with different substrate stiffness with normalized pre-stress of 10 × π^2^. When the pre-stress is chosen as 0.001 very close to zero, the deflection can nearly equal the results obtained without substrate [29], which can verify the accuracy of the analytical model in the post-buckling analysis. As substrate stiffness increases, the Young’s modulus of compliant substrate increases, and the deflection of the thin films decreases. When substrate stiffness changes from 0 to 500, the deflection of the film decreases from 4.9 to 3.0.

**Figure 3:**
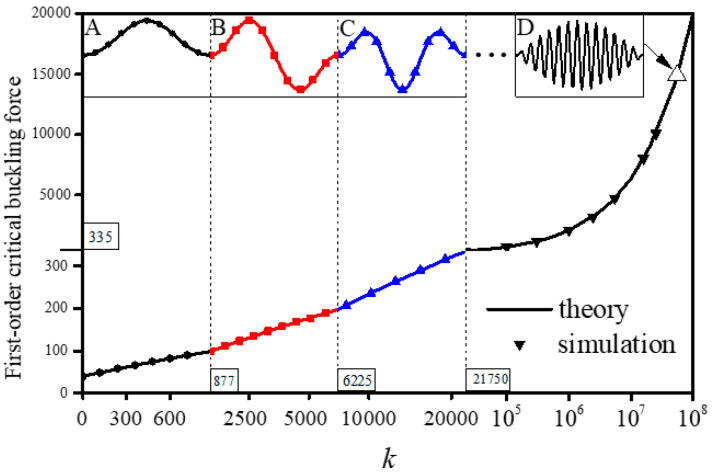
The first-order critical buckling force versus substrate stiffness comparison between theory and simulation when *P* = 10^6^. The first-order buckling mode with (**A**) *k =* 0~877, (**B**) *k =* 877~6225, (**C**) *k =* 6225~21,750, and (**D**) when *k =* 5.5 × 10^7^.

The first-order critical buckling force and the buckling modes can also be affected by substrate stiffness, as shown in Figure 3. When *k* is lower than 877, the buckling mode is shown in Figure 3A, but with increase of *k*, the first-order buckling mode gradually varies from global buckling to local buckling. The values of *k* at the transition points between the first fourth buckling modes are 877, 6225, and 21,750, respectively. Figure 3D exhibits the first-order buckling mode with *k* of 5.5 × 10^7^ where the number of wrinkles reaches up to 27, which is the typical morphing of local buckling. For comparisons, the number of wrinkles is just two or three when *k* belongs to the range of 877~6225 or 6225~21,750, respectively. The buckling modes and the first-order critical buckling force obtained from theoretical calculation agree reasonably well with simulation results.

**Figure 4:**
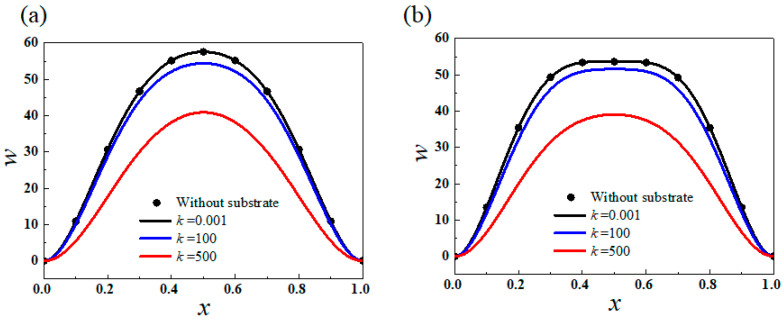
(**a**) The first order and (**b**) the second order vibration modes of the first-order buckling with different substrate stiffness or without substrate when *P* = 10 × π^2^.

Figure 4 illustrates the deflections of first and second vibration modes of the post-buckled thin film in the first-order buckling mode with compressive load of 10 × π^2^. When *k* is close to zero, the vibration mode can be validated by the results obtained by Ref. [29], and if *k* is larger, the deflections obviously decrease. When *k* increases from 0 to 500, the deflection of the first-order vibration mode decreases from 57.6 to 40.9.

**Figure 5:**
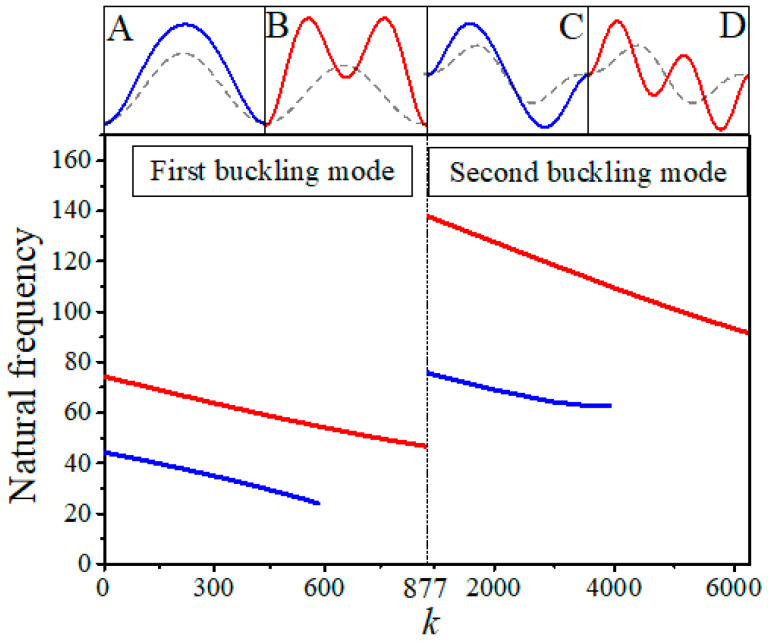
The first order (blue line) and the second order (red line) natural frequencies in the first two orders buckling modes with different substrate stiffness when *P* = 10^6^. (**A**,**B**) are the first order (blue line) and the second order (red line) vibration modes in the first order buckling mode (dot line). (**C**,**D**) are the first order (blue line) and the second order (red line) vibration mode in the second order buckling mode (dot line).

The substrate stiffness can have negative effects on the natural frequencies, which can be found in Figure 5. Blue lines and red lines in the main figure stand for the first and the second order natural frequencies for the system. However, the corresponding buckling modes are shown in the graphs A–D, where A and B show the vibration mode in the first buckling mode; C and D show the vibration modes in the second buckling mode. With increase of *k*, the first linear natural frequency and the second linear natural frequency both decrease gradually, and the first vibration mode disappears when the first nature frequency decreases to 24.13 below $\sqrt{k}$ in first buckling mode and decreases to 60.71 below $\sqrt{k}$ in second buckling mode, because Equation (22) turns into the condition $\omega^{2}>k$.

A typical stretchable electronics example, mechanical response of copper thin film on PDMS substrate subjected to a compressive load, is used to demonstrate the application of theoretical calculation. The buckling of copper thin film on PDMS substrate is widely used in various flexible electronic devices as the bridges in “island-bridge” [51,52]. The elastic modulus *E_s_*, the width *b*, the thickness *h*_s_, and the length *L* of PDMS substrate are 2 MPa, 4 mm, 1 mm, and 10 mm, respectively. The elastic modulus *E_f_* and the thickness *h*_f_ of copper thin film are 71,000 MPa and 0.01 mm. Meanwhile, the structure with fixed condition at both ends is subjected to a compressive load $\hat{P}$ = 2.367 × 10^−4^ N. The dimensionless quantities *k* and *P* are obtained by Equation (6),

(31) $$P=5000,\;k=3.38\times 10^{6}$$

**Figure 6:**
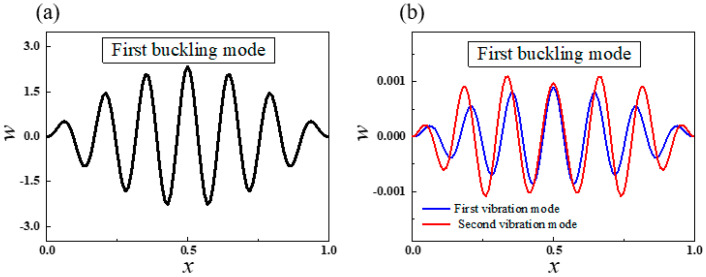
(**a**) The first order buckling mode and (**b**) the first order (blue line) and the second order (red line) vibration modes in the first buckling mode with substrate stiffness *k* = 3.38 × 10^6^ when *P* = 5000.

The mechanical response of this structure can be obtained by the theoretical analysis. The first order critical buckling force is 3677.2, and the first two orders natural frequencies in first buckling mode are 75.40 and 452.22. Figure 6 illustrates the first order buckling mode and its first two vibration modes. The wrinkle mode is helpful to understand the mechanical behavior of stretchable electronics [53,54].

## 5. Conclusions

In summary, an analytical model was developed for the dynamic behaviors of post-buckled thin films on a compliant substrate. The effects on buckling modes, buckling critical force, and vibration modes via stiffness of the substrate were investigated systematically. The increase of substrate stiffness can obviously reduce the deflection of post-buckling modes and vibration modes. Meanwhile, the first order buckling mode gradually varies from global buckling to local buckling, and the buckling mode is wrinkled. With the increase of substrate stiffness, the natural frequencies in the first two orders buckling modes drop noticeably. The results serve as guidelines for dynamic design of stretchable electronics to avoid resonance in a complicated noise environment, which is of great significance for the accurate measurement and the long-term use of flexible electronic devices [9,10,11,12,13,14] integrated on elastic substrate worked in complex service environments.
